# Piloting a digital campaign to promote awareness of the Louisiana TelePrEP program among sexual and gender minority young adults

**DOI:** 10.1371/journal.pone.0290149

**Published:** 2023-08-29

**Authors:** Manuel A. Ocasio, M. Isabel Fernandez, Steven Cortese, Kathryn Kampa

**Affiliations:** 1 Department of Pediatrics, School of Medicine, Tulane University, New Orleans, Los Angeles, United States of America; 2 College of Osteopathic Medicine, Nova Southeastern University, Fort Lauderdale, Florida, United States of America; 3 Communify, New Orleans, Los Angeles, United States of America; 4 Department of International Health and Sustainable Development, School of Public Health and Tropical Medicine, New Orleans, Los Angeles, United States of America; Human Sciences Research Council, SOUTH AFRICA

## Abstract

Despite the proven efficacy of pre-exposure prophylaxis (PrEP) in preventing HIV acquisition, PrEP is underutilized by sexual and gender minority young adults in the 13 states in the United States “South.” In this paper, we describe the process we used to develop a digital campaign to promote awareness and engagement of sexual and gender minority young adults in the Louisiana Department of Health’s TelePrEP Program and provide campaign performance metrics. In Phase 1, we conducted formative research that informed campaign development and strategy. In total, 109 sexual and gender minority young adults completed a survey of PrEP constructs (e.g., facilitators, barriers). We also conducted three, sequential focus groups to iteratively generate, revise and refine the digital material. In collaboration with our strategic marketing partner (SMP), we developed 3 different ads and 1 video ad to promote on web and mobile-in app display, as well as Facebook, Instagram, and YouTube. Phase 2 focused on campaign implementation and evaluation (e.g., number of impressions, user activities on LA TelePrEP landing page). In the first few weeks of the campaign, data from tracking pixels indicated minimal activities on the landing page. We paused to revamp the campaign. Our SMP determined that a more young adult-focused landing page could bolster engagement. We created a new landing page and reran the campaign for 33 days. We saw substantially more user activities on the new landing page (n = 382) compared to the LA TelePrEP landing page (n = 185). Overall, we had 730,665 impressions and 475 link clicks. By collaborating with our SMP, we effectively and efficiently translated our community-engaged formative research into relevant and engaging digital content. This pilot study is one of the first to demonstrate the importance of using tracking pixels to monitor real-time user data to optimize performance of a digital PrEP campaign.

## Introduction

Despite the advances of HIV prevention efforts in many areas of the United States, Louisiana and the other 13 states (Texas, Oklahoma, Arkansas, Mississippi, Alabama, Georgia, Florida, North and South Carolina, Tennessee, Kentucky, Virginia, West Virginia) referred to as the “South” in the HIV surveillance reports continue to be heavily impacted. The “South” accounted for 51% of the new HIV diagnoses in 2020 [1]. Furthermore, the “South” has the highest rate of HIV in rural areas and fewer people who are aware of their HIV status compared to other regions of the country. Sexual minority young adults (persons with non-heterosexual identity, attraction, and/or sexual contact) and gender minority young adults (persons whose gender identity or expression is different from their assigned sex at birth) [2] in the “South” are at particularly elevated risk relative to their cisgender heterosexual counterparts. In the “South”, cisgender gay, bisexual and other men who have sex with men, particularly younger men, bear the brunt of the HIV epidemic [3]. Limited data also suggest that 14% of transgender women and 2% of transgender men are living with HIV, a majority of whom are in the “South” [1]. In Louisiana, adolescents and young adults 13 to 24 years of age comprised 21% of all new HIV cases diagnosed in 2019. Almost half of these new cases were concentrated in New Orleans and East Baton Rouge [4].

Given these sobering statistics, it is not surprising that approximately half of all jurisdictions in the Ending the HIV Epidemic initiative (EHI) are situated in the “South”. The EHI is a U.S. Department of Health and Human Services initiative designed to reduce new HIV infections by scaling up HIV prevention and treatment strategies in the areas with the highest concentration of new HIV cases diagnosed [5]. To achieve the target of 90% reduction of new HIV infections by 2030 [5], prevention efforts must be ramped up in EHI jurisdictions so that those most at-risk can access and utilize effective HIV prevention methods.

Pre-exposure prophylaxis (PrEP) is a potent and efficacious strategy to prevent HIV acquisition. PrEP has been found to effectively reduce HIV transmission in a number of groups from diverse geographic areas including adult cisgender gay, bisexual and other cisgender men who have sex with men, people of transgender experience, heterosexual men and women, and serodiscordant couples [6]. The efficacy of PrEP, when taken consistently, has also been demonstrated among young cisgender gay, bisexual and other men who have sex with men [7, 8]. When taken as prescribed, PrEP can reduce the risk of getting HIV from sex by about 99% [9]. Yet, PrEP is underutilized particularly by racial and ethnic sexual and gender minority adolescents and young adults from EHI jurisdictions in the “South” [10] such as New Orleans and East Baton Rouge, Louisiana. For instance, in 2021, the PrEP-to-Need Ratio (PNR), a metric that reflects whether PrEP use appropriately reflects the need for HIV prevention, was lowest among young people aged 13 to 24 relative to all other age groups in Louisiana [11]. Stigma related to HIV and PrEP use have been consistently cited as barriers to PrEP uptake [12, 13]. A 2022 study of Black and Latinx sexual minority cisgender men and transgender women found that PrEP stigma was independently associated with current PrEP use; however, when researchers tested for the moderating effect of logistical barriers, PrEP stigma was only significant when logistical barriers to PrEP use were low [14]. Thus, programs that can reduce logistical barriers to PrEP access, while addressing related stigma among sexual and gender minority young adults are needed to increase PrEP use.

To increase PrEP access, the Louisiana Department of Health launched the Louisiana TelePrEP Program (LA TelePrEP) in 2018. This novel telemedicine program connects Louisiana residents 18 years of age or older to a provider who prescribes PrEP and to an e-navigator who facilitates enrollment in the program, lab testing, insurance access, and other ancillary services. Enrollment can be done remotely by completing the form accessible on the LA TelePrEP website [15]. Telemedicine approaches can reduce stigma which is often experienced in face-to-face clinical settings and eliminate commonly cited logistical barriers, such as transportation [16]. Unfortunately, relatively few sexual and gender minority young adults have enrolled in the program despite outreach and promotional efforts launched at local colleges, distribution of flyers at community-based organizations (CBOs) and clinics, and posts through the health department’s social media profile. Clearly, the department’s efforts were not reaching young adults who are sexual and gender minority; alternative strategies for increasing the sexual and gender minority young adult’s participation in the TelePrEP program were sorely needed.

In 2019, the Adolescent Trials Network for HIV/AIDS Interventions (ATN) published a call for applications with a short project period to advance the goals of the EHI targeting adolescents and young adults. Partnering with health departments and/or community-based organizations (CBOs) was a cornerstone of the call given the pivotal role these entities play in the fight against HIV. In response to this call, ATN affiliated researchers from two universities in the South partnered with the Louisiana Department of Health to develop and pilot a digital campaign to increase awareness of the LA TelePrEP program among young adults who are sexual and gender minority 18 to 29 years of age.

Digital technologies offer great promise for increasing awareness of the LA TelePrEP program among sexual and gender minority young adults. Ninety percent of young people regularly use digital technologies (e.g. social media) to gain and share information, post pictures, conduct financial transactions, stay current and build social connections [17, 18]. In addition to being accessible, familiar and user-friendly, online tools available on many social media sites enable the direct purchase of banner ads [19], at reasonable and affordable price points. These ads could be used for a variety of purposes including providing information about programs and services, directing users to different landing pages, and prompting users to take specific actions (e.g., complete a screener). Furthermore, many digital platforms such as Google, Facebook and social media apps collect data on users’ interest and online behavior. These data can be used to target specific subpopulations, such as young adults who are sexual and gender minority in Louisiana, efficiently and cost-effectively as well as direct ads to individuals who are most likely to engage with them [19, 20]. It is not surprising that there has been a dramatic increase in the use of technology for HIV prevention research and practice.

Given the challenges of engaging hard-to-reach populations such as young adults who are sexual and gender minorities, significant attention has been focused on social media and dating app-based advertisements to increase awareness of on-going research studies and enhance recruitment [21–23]. These studies provide strong evidence that digital ads can be used to encourage sexual and gender minority young adults to engage with research studies by completing the screening questionnaires. Many of these studies use impressions, clicks, click through rate (CTR) and cost per click as evaluation metrics. Until recently, relatively little attention has been given to comparing across different social media apps and platforms. Zlotorzynska, Bauermeister, Golinkoff et al [23] used engagement, screening and cost metrics to compare the performance of ads placed on Facebook, Instagram, Snapchat, Twitter and Grindr to recruit young adults who are sexual and gender minorities across the United States. They found considerable differences in performance across the platforms. For instance, Instagram proved most effective for reaching young, eligible racial and ethnic minorities. The lowest cost per eligible youth were ads placed on Facebook properties. Furthermore, there were regional differences in cost per eligible contact.

Dehlin, Stillwagon, Pickett et al [24] reported on a grassroots effort to develop and launch a multi-media campaign that included billboards, posters and other print ads as well as digital media outlets to promote PrEP awareness and use among sexually-active people in Chicago. Advertisements directed users to the campaign website and a hotline for a warm handoff to PrEP services. They evaluated the performance of both the print and the social media ads during a four-month period. Across social media platforms and display advertising, their ads were viewed 40 million times and the campaign website averaged 182 clicks per day. They estimated that 83 people who saw the ads contacted the hotline and 18% started PrEP. The successful use of digital media ads for awareness and engagement, support developing a targeted digital campaign to increase awareness of the TelePreP program among young people who are sexual and gender minorities in Louisiana. In this paper, we describe the process we used to develop the digital campaign in hopes that others could use our experiences to guide their campaign development efforts. Since the goals of our modest campaign were to increase awareness of the TelePrEP program and engagement with the campaign landing page, we provide campaign performance metrics to illustrate how the campaign impacted user awareness and engagement.

## Methods

In Phase 1, the formative research phase, we conducted a community survey and sequential focus groups that guided and informed the development of the campaign material and strategy. We partnered with a locally based LGBTQ+ strategic marketing agency. We also designed the protocols for launching the campaign and defined the metrics we would use to evaluate its reach. The materials, protocols and metrics were developed iteratively with extensive input from our partners and community members. In tandem, we iteratively developed a chatbot that would support the e-navigator and disseminate information about PrEP and the TelePrEP program. We describe our chatbot development protocol in a separate manuscript [25]. Phase 2 focused on implementation and evaluation of the digital campaign and launch of the chatbot. The project period was 20 months.

The study was approved by the Tulane University Institutional Review Board (2021–107). Only IRB-approved authors and study staff had access to identifiable participant information that was collected during Phase 1 formative research. These data will be destroyed once study dissemination activities are completed.

### Phase 1

#### Community survey

We developed and programmed a brief Qualtrics survey to identify key indicators that capture constructs related to PrEP uptake (experiences, intentions, beliefs, facilitators, barriers) in sexual and gender minority young adults. The majority of the survey items were drawn from the published literature and reviewed by our Youth Advisory Board, which consisted of 8 young adult members (88% ethnoracial minority, 100% sexual or gender minority). We used both passive and active recruitment methods. Active recruitment included directly approaching youth at community events, bars and clubs frequented by sexual and gender minority young adults and inviting prior study participants who had consented to be contacted for future studies. Participants were invited to refer others to the study. Additionally, Louisiana Department of Health and sexual and gender minority-serving community-based organization (CBO) staff shared recruitment materials with clients and encouraged them to participate in the study. For passive recruitment, we posted flyers at community venues frequented by sexual and gender minority young adults and paid advertisements on Instagram, Facebook, and Jack’d, a popular gay dating app. We also used a study-specific profile to disseminate the survey on social media sites. Young adults who completed the survey received $10 and an invitation to participate in the focus group component. Participants were compensated $5 for each person they referred who completed the study. All compensation was distributed via CashApp. We implemented multiple strategies to identify fraudulent entries, such as survey bot responses and duplicate entries. First, we created separate links for the screener, community survey and payment form to minimize the ability for a bot to complete all forms. Second, we programmed the survey to limit one response per IP address. Third, we used timestamps to determine how close in time consecutive surveys were completed and the length of time for survey completion to differentiate rapid responses by a bot from a human being.

From June, 2021 to March, 2022 we enrolled 109 young adults who: 1) self-identified as sexual and/or gender minority; 2) were 18 to 29 years of age; 3) reported condomless sex in the last 6 months; 4) lived in Louisiana; 5) and were HIV negative or unknown HIV status. Thirteen participants were recruited through referrals. On average, participants were 22 years of age; 85% came from the Greater New Orleans area and 7% came from East Baton Rouge. Sixty-two percent identified as cisgender men, 54% as gay and 26% as gender diverse (e.g., non-binary, genderqueer); and almost half (47%) were Black or African American. Although 89% of the sample had heard of PrEP, a majority had never taken PrEP (63%). Participants reported that the main facilitators of using PrEP were that it protects against HIV (55%), is easier to use than other methods (32%), and does not interrupt sex (31%). In contrast, the main barriers identified were cost (31%), having to take a daily pill (53%), and the potential for harmful side-effects (38%). Awareness of the TelePrEP program was low; only 25% of the sample knew about the program. A majority of participants reported using Instagram (86%) and 40% reported using Instagram more than any other social media apps.

#### Focus groups

Guided by the survey results, we developed a focus group discussion manual that tapped the following areas: 1) social media use (likes, dislikes); 2) perceptions of HIV-related messaging on social media; and 3) recommendations for promoting TelePrEP on social media. We convened three focus groups on HIPAA-compliant Zoom that were conducted from April to July, 2022. Each focus group was comprised of 4–6 participants who had completed the on-line survey and was audio-recorded with permission. Participants received $40 as compensation via CashApp. All participants provided written consent electronically.

Focus groups were led by two trained facilitators. Participants viewed sample digital material and used a five-point scale (1 not at all 5 extremely) to anonymously rate the content of each sample as being: 1) engaging; and 2) convincing. After reviewing the responses for each component, participants discussed their ratings, what guided their decisions, and provided recommendations for improvement. We were especially interested in understanding participants’ thoughts on visual (imagery, colors, font) and textual (tone, language, clarity) aspects of the content. Facilitators independently took notes during focus groups and reviewed recordings to modify notes, as needed. The focus groups followed the same procedures, although the sample digital content presented differed.

The content of the focus groups was developed in collaboration with our strategic marketing partner (SMP) and followed an iterative process to generate, revise and refine the digital material. For the first focus group, we presented digital material from existing PrEP-related promotional materials and selected the most salient PrEP related messages based on the survey result. We also included existing LA TelePrEP advertisements. The results of the first focus group were used to create the digital content for the second focus group and the results of the second focus group generated the content for the third focus group. The materials were again refined prior to being presented to the Louisiana Department of Health Material Review Committee and Bureau of Media and Communications for final review and approval. This process yielded the three digital advertisements (and banner ads) used in the campaign.

**Fig 1:**
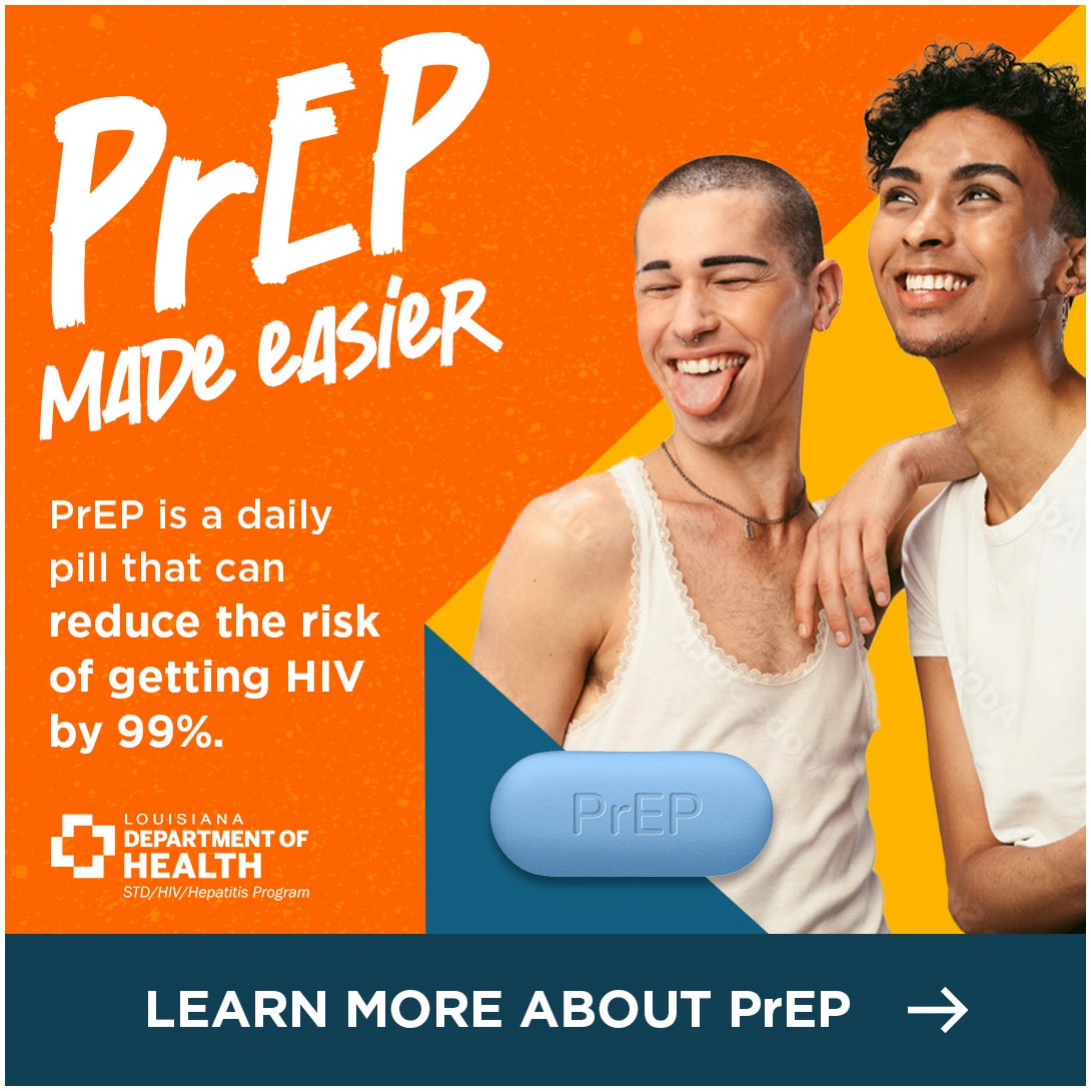
Version A of the TelePrEP campaign ad.

**Fig 2:**
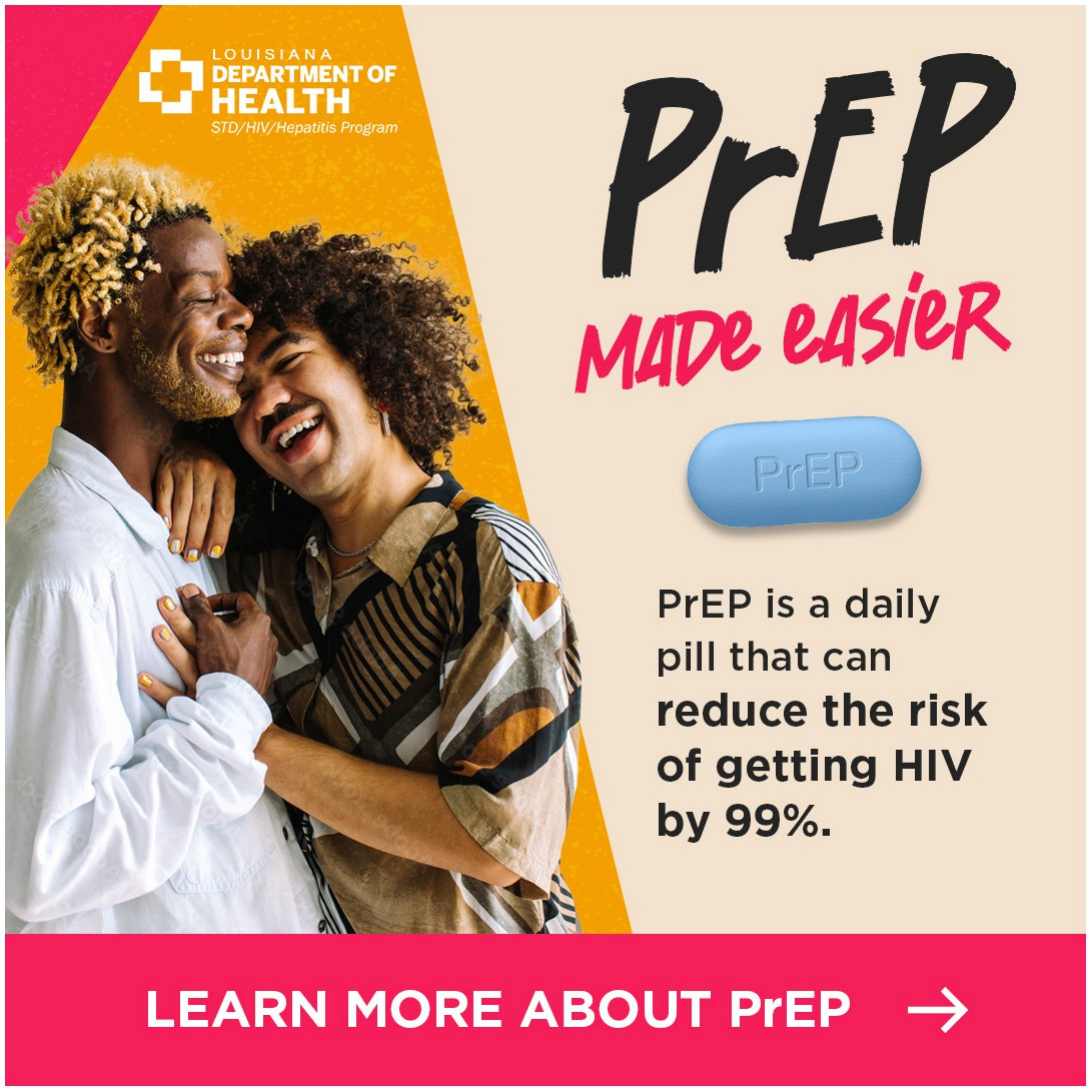
Version B of the TelePrEP campaign ad.

**Fig 3:**
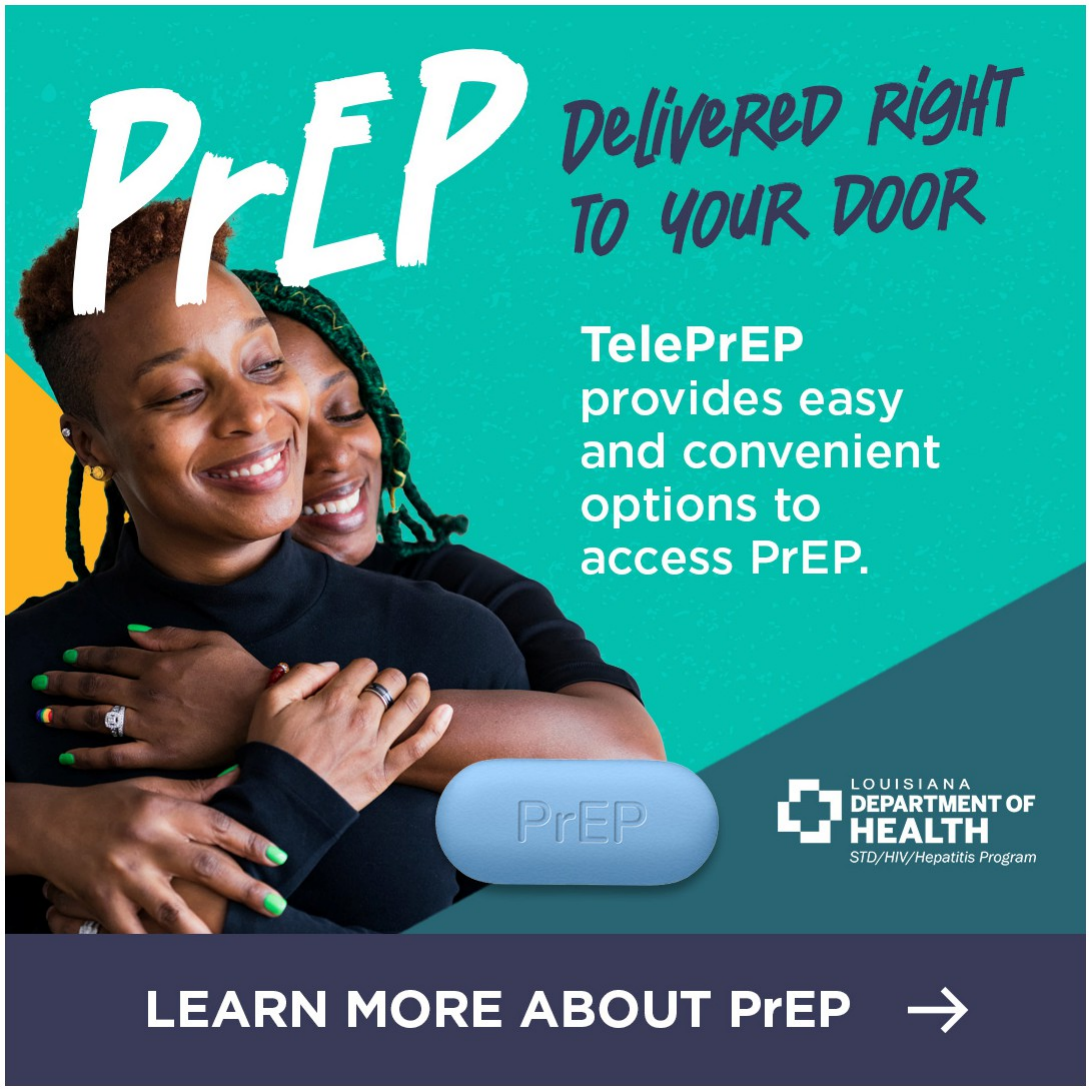
Version C of the TelePrEP campaign ad.

We identified consistent themes across the 3 focus groups that informed the final digital campaign creative and strategy. In terms of content, participants recommended that the materials include factual information about PrEP’s efficacy and having an imprimatur from a known and credible organization such as the health department. Participants recommended highlighting aspects of the TelePrEP Program that would resonate with sexual and gender minority young adults such as privacy and home delivery. In terms of visuals, participants strongly preferred ads with more than one racially diverse, overtly queer young adult over those with infographics or text only. They encouraged us to use attention grabbing bright colors, motion and music and to advertise on social media. We considered these recommendations as we developed each iteration of the digital materials and the digital campaign strategy. In total, we developed 3 ads (Figs 1–3).

#### Digital campaign strategy

Armed with the results from the community survey (salience of PrEP efficacy, low awareness of TelePrEP program, social media preferences) and focus groups (bright colors, overt queerness, privacy, home delivery), we worked collaboratively with our SMP to define the goal of the digital campaign and develop a marketing strategy that would appeal to sexual and gender minority young adults. The primary goal of our digital campaign was to raise awareness of the TelePrEP program. We arrived at this decision because almost three-fourths of survey participants had not heard about the TelePrEP program and few sexual and gender minority young adults had enrolled in the TelePrEP program. We delineated the key performance indicators (KPI) for awareness as: 1) exposure measured through number of impressions; and 2) interactions measured as number of clicks and click-through rate (CTR). Even though the short project period limited the duration of the digital campaign, we set a secondary, exploratory goal of engagement with the campaign website to inform future efforts. To track engagement, we used two metrics: 1) the number of visits to the landing page (the Louisiana Health Hub’s TelePrEP Program landing page) resulting from exposure to our ads; and 2) user activities on the landing page. We placed tracking pixels on the LA TelePrEP landing page to log number of visits to the landing page, which advertising strategy led users to the landing page, and track user activities on the landing page. In addition to these performance indicators, we also tracked cost efficiency of each digital strategy (i.e., cost per click). See Table 1 for a description of metrics and key terminology.

**Table 1:** Descriptions of key terms.

Term	Definition
**General Terms**	
Tracking Pixel	Code embedded in a website (e.g., landing page) to track actions users take on a website.
Call to Action	Text or visual element that prompts a desired action.
Impressions	Number of times an advertisement was viewed (not unique users. One user can contribute to impressions more than once)
Link Clicks	Number of times a person clicked on the call to action button that leads to the website (e.g., landing page).
Click-Through Rate	Percentage of users who click on an advertisement (to enlarge, call to action to the number of impressions.
Link Click-Through Rate	Percentage of users who clicked on link to website (e.g., landing page) to the number of total number of impressions
Cost Per Click	Cost allocated to digital strategy (e.g., display) divided by number of link clicks.
Cost per Impression	Cost allocated to digital strategy (e.g., display) divided by number of impressions.
Site Activity	Prespecified actions that are tracked on a website (e.g., landing page).
**Display Advertising Terms**	
Contextual Targeting	Users identified based on presence of a keyword on a website they are visiting for future advertising.
Search Targeting	Users identified based on use of keywords in a search engine.
Mobile In-App Advertising	Ads displayed in mobile apps.
Extended Audience	Campaign feature that uses machine learning to identify similar users from the keyword list.
**Social Media Advertising Terms**	
Awareness Objective	Objective to maximize reach to users who are most likely to remember ad.
Traffic Objective	Objective to drive traffic to the website (e.g., landing page).
**YouTube Terms**	
Video Views	Number of users that viewed a video (equivalent to impressions).
Video Completion Rate	Percentage of users who viewed a video to the end.
Topic	Targeting method to show users video based on type of viewing content.
Audience	Targeting method to show videos based on user characteristics (interests, demographics)

We decided to use display ads (web and mobile in-app) as well as ads posted on Facebook and Instagram since 86% of survey participants used Instagram and 66% used Facebook. To direct the placement and management of our display ads, we used the Google Ads advertising platform which provides a streamlined process for setting up a targeted digital campaign via search, contextual targeting and audience expansion algorithms (Google support) guided by a keyword list. For ads on Facebook and Instagram, we used Meta Ads Manager and selected awareness and traffic as our goals. Our target audience settings were set to target 18 to 24 year olds in zip codes with the largest population density in Louisiana [26]. We used Meta’s algorithm, rather than manual selection, to optimize ad placement. Thus, as a campaign runs, Meta will place more ads on the platform that is performing better.

### Phase 2

In September of 2022, we launched the digital campaign. The SMP monitored the campaign’s performance and provided weekly updates. Three weeks into the campaign, KPI data showed a high number of impressions (exposure) and large numbers of link clicks (interactions). However, the tracking pixel data demonstrated minimal engagement with the campaign’s landing page. Though engagement activities were exploratory, we were concerned that there was such dramatic inconsistency between ad engagement and website use. After 28 days, we paused the campaign to identify potential reasons for the lack of engagement and develop viable solutions. First, we evaluated the characteristics of the landing page itself. In contrast to the bright colors (bright orange, pink, turquoise) and youthful font of our ads, the TelePreP Program’s landing page used more subdued colors (muted green and blue) and a more generic font that matched those used by the Louisiana Department of Health. Thus, the stark visual contrast between our ads and those on the landing page may have turned off users. Second, we examined the content. Rather than a direct and focused “call to action”, the landing page offered users multiple “calls to action”, which may have led users to feel overwhelmed, distracted and unwilling to select an option. For example, the landing page had comprehensive information on the program as well as provided options to schedule an appointment, text the e-navigator, and read FAQs. Third, we explored the feasibility of developing video content as recommended by focus group participants.

Based on this evaluation, we paused the campaign for 33 days and revised the digital strategy. We created a campaign-specific landing page that mirrored the colors and font used in our digital ads. The only call to action was directing users to text an SMS chatbot for more information [25]. Because we lacked sufficient resources and time to create a storyboard for the video and film its content, our SMP created a motion graphic video maintaining the campaign aesthetic and incorporating suggestions from our focus groups. We added YouTube as a display medium for our video, because of its popularity among young people [27]. The video ad that ran on YouTube can be viewed at: https://youtu.be/1FMKpB2m5TQ. The revised campaign ran for 40 days.

## Results

Over the course of the campaign, we had more than 730,000 impressions and 475 link clicks. Table 2 shows data comparing KPI and cost efficiency across our digital advertising strategies. Facebook was the most efficient in terms of CTR (0.14%) and combined with Instagram, had the lowest cost per click ($8.62). Notably, Facebook outperformed Instagram, which was the most popular app among our study participants, in number of impressions and CTR. Web display advertising had the highest number of impressions (n = 458,172) and yielded the most link clicks overall (n = 278). Though YouTube was the least cost effective and had the fewest number of link clicks, there were 39,364 complete video views for a 66.6% video completion rate.

**Table 2:** Key performance indicators and cost efficiency across strategies.

Strategy	Impressions/Views	Link Clicks	CTR	Cost Per Click
**Facebook**	**108,105**	**149**	**0.14%**	**8.62** ^a^
**Instagram**	**40,407**	**11**	**0.03%**	
**Web Display**	**458,172**	**278**	**0.06%**	**$ 8.89**
**Display In-App**	**64,859**	**22**	**0.03%**	**$ 22.73**
**YouTube**	**59,122**	**15**	**0.03%**	**$ 200.00**

^a^Cost per click could not be separated by platform.

Table 3 summarizes the data generated by the tracking pixels. Over the course of the campaign, we tracked a total of 567 engagement activities across both landing pages. There was more than double the number of pixels fired on the new campaign-specific landing page when compared to the TelePrEP landing page even though the duration of the campaign periods were comparable. Beyond opening the website and allowing it to load, there was more engagement with the new landing page (viewing and clicking on “Learn More”) relative to the TelePrEP landing page. Other calls to action that we tracked (e.g., scheduling a consultation and calling or texting the Program) had zero engagement by users and thus are not shown on the table.

**Table 3:** User activities on the TelePrEP landing page and new campaign-specific landing page.

Website Activity	Description	Total Activities
LA TelePrEP Landing Page		185
Landing Page	A user lands on the website and it loads.	165
PrEP Directory	A user opens the PrEP Directory page.	5
Learn More (Click)	A user clicks on the “LEARN MORE” button.	14
Click Here to Enroll (Click)	A user clicks on the "CLICK HERE TO ENROLL" button.	1
New Landing Page		382
Landing Page	A user lands on the website and it loads.	215
Learn More (Visibility)	A user scrolls down and the "LEARN MORE" button is visible.	104
Learn More (Click)	A user clicks on the "LEARN MORE" button.	42
Text Our PrEPBot (Click)	A user on a mobile device clicks on "TEXT OUR PREPBOT".	21

Table 4 compares the performance of the ads. While KPIs for ads on web display was comparable (all 0.12% CTR), CTR varied in social media advertising. Overall, Facebook had higher CTR compared to Instagram and web display, specifically Versions A (CTR = 0.24%) and B (CTR = 0.23%). Version A delivered the most web activity (n = 191) on web display, while Version B delivered the most web activity on social media platforms (n = 36).

**Table 4:** Metrics for key performance indicators for different ad versions across social media platforms and display advertising.

	Social Media							Display			
	Facebook			Instagram			All Social Media^a^				
Ad Version	Impressions	Link Clicks	CTR	Impressions	Link Clicks	CTR	Any Web Activity	Impressions	Link Clicks	CTR	Any Web Activity
Version A	11,388	27	0.24%	4315	3	0.07%	11	152,590	176	0.12%	191
Version B	42,124	95	0.23%	16459	5	0.03%	36	151,479	189	0.12%	153
Version C	54,593	27	0.05%	4315	3	0.07%	2	151,269	187	0.12%	118

^a^Web activities could not be disaggregated by social media app.

## Discussion

The goal of our study was to develop and pilot a digital media campaign to increase awareness and engagement of sexual and gender minority young adults in the Louisiana TelePrEP program. Our study highlights the benefits of engaging community members and digital marketing experts in all phases of the campaign development and implementation process. In a rapidly evolving digital environment with changing trends and constant barrage of information, advancing digital-based HIV prevention efforts for sexual and gender minority young adults requires technical knowledge and expertise that few scientists possess. Researchers must expand their collaboration beyond traditional scientific disciplines to the business and technology sectors and partner with digital marketing experts such as our SMP. By collaborating with our SMP, we effectively and efficiently translated our community-engaged formative research into relevant and engaging digital content. Furthermore, by using tracking pixels to monitor campaign activities in real time, our SMP detected a problem with user engagement within 3 weeks of the launch and guided us to make the necessary adjustments to improve engagement, rapidly and effectively.

The strategy of using tracking pixels to monitor user behavior on the landing page in response to our ads is an important contribution suggested by our SMP that has not been frequently used in HIV prevention campaigns for sexual and gender minority young adults. Tracking pixels are a powerful tool that can be used to directly compare KPIs across digital platforms and different forms of advertising to evaluate progress towards campaign goals in real time. In our pilot, using tracking pixels to monitor campaign performance yielded the real-time data needed to refine our campaign strategy. Having performance data early proved particularly useful in our situation given our short project period where time was of the essence and our resources were limited.

Despite the benefits, tracking pixels do not provide information on the personal characteristics of the users; they only monitor user activity. Thus, we could not directly ascertain that all users tracked were sexual and gender minority young adults. Our digital campaign focused on increasing awareness of the TelePrEP program and user engagement with the landing page and not on recruiting eligible individuals to complete on-line surveys or participate in intervention trials. When social media is used as a recruitment strategy, the data to ascertain the demographic profiles of users is available from completed screeners, surveys or other measurement instruments [23, 28, 29]. To report with certainty that all of our users were sexual and gender minority young adults, we would have had to add a brief survey as a call to action on the landing page which was beyond the scope of our efforts. Because the content (e.g., the images, the poses, the captions) and placement/targeting of the ads were designed to appeal to sexual and gender minority young adults, it is likely that, at minimum, a proportion of our users were sexual and gender minority young adults. Other researchers’ success in reaching and enrolling young sexual and gender minorities into studies using similar targeting approaches to ours [23], supports this perspective.

Our experience with tracking pixels has implications that extend beyond digital campaigns. Health promotion interventions and programs are increasingly using digital technologies [30, 31]. For instance, apps are being used as delivery vehicles [32], users are being referred to websites for information and resources [33]; and services are being advertised through social media. Embedding tracking pixels into health promotion interventions and programs holds tremendous promise for helping researchers and practitioners gain important data to make rapid adjustments and improve performance. However, this area is not without controversy as data privacy has been a primary concern in digital advertising. Industry and government have been gradually setting policies and regulations to limit tracking specific individuals. For example, Apple has instituted opt-in permissions for third-party apps to access user data [34]. In our case, our SMP did not collect identifiable information; we only collected data pertaining to user engagement on the landing page. Furthermore, in keeping with the increasing trend for respecting user privacy, our partners maintain strict requirements with marketers to ensure that they will not use any user data without user consent. This reinforces the importance of collaborating with partners that have strong policies to protect against misuse of data.

In addition to monitoring user activities, it is important to examine the performance of the digital content per se and the placement of the content. Monitoring these parameters can yield real-time information to refine campaigns such as revising or removing lower performing content in favor of the better performers or shifting the placement of the ads. We saw no difference in CTR among the three versions of the digital content when they appeared as display ads on websites or apps. Yet in social media, Versions A and B outperformed Version C.

The performance of the YouTube video is worth noting even though the results should be interpreted with caution because of the limited time that the video was shown. Industry standards vary in terms of what constitutes a reasonable video completion rate, but some consider 15% to be acceptable [35] and 70–80% as good [36]. Our 66.6% rate falls on the higher end of this spectrum, suggesting that YouTube is promising for disseminating information. In terms of ad placement on social media platforms, we were surprised to find that Facebook yielded better KPIs for campaign awareness than Instagram. Results from our community survey and nationally-representative surveys indicate higher Instagram use among young adults compared to other social media apps [37]. Furthermore, social media marketing experts recommend Instagram over Facebook for effective brand awareness among younger populations [38]. However, because our campaign period was brief, we cannot definitively claim Facebook as a more effective platform for promoting awareness of TelePrEP programs among sexual and gender minority young adults. If we had had a longer project period, we would have enhanced our presence on social media and YouTube and made adjustments on placement for the ads. Additionally, we would have reduced the budget for display ads, particularly for mobile in-app display. It is likely that these revisions would have yielded better performance at the same or reduced cost. Having real-time data during implementation can be extraordinarily helpful to improve campaign performance as our findings have shown.

Despite the novel findings, there were some limitations to our study. As with other digital campaigns, we did not have a way to ascertain the exact demographic profile of users who viewed and engaged with ads and the landing pages. Thus, we cannot be certain that our users were actually young sexual and gender minorities even though we designed the campaign to target them. Furthermore, the short timeframe and small budget significantly limited our ability to more fully evaluate the performance of our digital campaign. Use of both print and social media ads over a longer period would have enhanced the performance reaching more users. Research has shown that multiple exposures to ads via different outlets is a key element for effective health communication campaigns [39]. With more resources and time, we could have maximized reach by targeting other social media apps, as well as popular gay dating apps that have successfully engaged youth in HIV prevention research [23, 40]. Lastly, we would have worked with the Louisiana Department of Health to design a TelePrEP website tailored to youth that would increase engagement. Notwithstanding the limitations of our study, our campaign increased awareness of PrEP among some users. Because PrEP awareness and uptake is considerably low among key populations (e.g., heterosexual Black women) especially in the South, any effort that helps to increase awareness is beneficial.

This pilot study is one of the first to demonstrate the importance of using tracking pixels to monitor real-time user data to optimize performance of a digital PrEP campaign. The feedback and recommendations provided by our SMP led us to make immediate adjustments that improved engagement. The rapid pace in which digital technologies advance necessitates strong partnerships between public health practitioners and technical experts. By so doing, we will be able to fully realize the power of digital ads to advance HIV prevention efforts for young sexual and gender minorities in the South.
